# Level of quality of option B^+^PMTCT service provision in public health facilities in Mekelle zone, northern Ethiopia: cross-sectional study

**DOI:** 10.1186/s12913-020-05429-6

**Published:** 2020-06-17

**Authors:** Kiros Fenta Ajemu, Alem Desta

**Affiliations:** 1 Tigray Health Research Institute, Mekelle, Tigray Ethiopia; 2 School of Public Health, Mekelle University, Mekelle, Tigray Ethiopia

**Keywords:** Quality, Option B + PMTCT, HIV positive women, Tigray, Northern Ethiopia

## Abstract

**Background:**

Substantial improvements have been observed in coverage and access to maternal health services in Ethiopia. However, the quality of care has been lagging behind. Therefore, this study aimed to assess the level of quality of Option B^+^ PMTCT in Northern Ethiopia.

**Methods:**

A facility based survey was conducted from February to April 2016 in Northern Ethiopia. Twelve health facilities were enrolled in the study. Mixed method approach was used in line with Donabedian (Input- Process-Output) service quality assessment model. Data of 168 HIV positive mothers & their infant were abstracted from registers, and follow up charts. During the Option B+ service consultation, a total of 60 sessions were involved for direct observation. Of which, 30 clients and 12 service providers were subjected for exit and in-depth interview respectively. Facilities were categorized rendering good service quality based on predetermined quality judgment criteria. Reasons of good and bad service quality were thematically fitted with each quality component based on emerging themes (TM1-TM3), and categories (CA1-CA6).

**Results:**

Of the total 12 study health facilities, 2(16.7%) were achieved the desired level of service quality based on the three quality components. The input quality was better and judged as good in 33.3% health facilities. However; process and output service quality were realized in one - fourth of them.

**Conclusion:**

Insignificant numbers of facilities fulfilled the aspired level of service quality. Quality of care was found influenced by multiple inputs, processes, and output related barriers and facilitators. Comprehensive Program monitoring is needed based on three quality components to improve the overall service quality.

## Background

Globally, Mother to Child Transmission (MTCT) of HIV accounts for 90% of new pediatric HIV infection worldwide. Sub-Saharan Africa experienced the greatest share [1]. In order to increase the likelihood of children being born free from HIV, World Health Organization (WHO) had been implementing different strategies (Option A, Option B, Option B^+^) for optimizing PMTCT care and support for low and middle income countries since 2001 [2]. Under Option A, women receive antepartum (starting at 14 weeks of gestation) and intrapartum ARV prophylaxis to reduce the risk of drug resistance. Meanwhile, women receive triple ARVs starting as early as 14 weeks of gestation and this is continued through childbirth if not breastfeeding or until one week after the cessation of breastfeeding in cases of Option B^+^ in MNCH Platform [2, 3].

During PMTCT scale up period (Option A, & B), significant achievements were obtained in rolling back new pediatric HIV infection by 70% [3]. Nevertheless; programmatic, clinical, and other operational challenges were reminded that lead high attrition and lost to follow up of HIV positive pregnant and lactating women from HIV chronic care. In 2010, an estimated 23% of HIV positive women lost to follow up and nearly 150,000 new pediatric HIV infections were reported worldwide [1–3]. Besides, fewer than 20% of HIV infected pregnant women received any PMTCT intervention and as many as 14% of infants bore from HIV infected women were tested positive for HIV in Malawi [4]. The high attrition rate of HIV positive women from HIV chronic care was reported from Sub-Saharan Africa (28%) and Cameroon (24.3%) [3–5].

It had been a great challenge in Ethiopia in which only 10% of HIV positive pregnant and lactating women complete PMTCT program until infant HIV confirmatory testing at 18 months of age [6]. In the early 2013, a third option (Option B^+^) has advocated to provide lifelong first line regimen of tenofovir/lamivudine/efavirenz (TDF/3TC/EFV) to all HIV infected pregnant and lactating women regardless of CD4 cell count [7]. This new alternative approach has programmatic and clinical advantage in which it is fully integrated in to MNCH platforms, test and treat strategy regardless of CD4 count that could accelerate progress towards eliminating new pediatric HIV infections [8]. This programmatic update also emphasized additional advantages such as providing better protection for maternal health and reduction in sexual transmission of HIV [7, 8].

Option B+ was first conceived and implemented in Malawi. Preliminary findings of routine Option B+ PMTCT programme in a rural district in Malawi showed a fivefold increase in ART initiation and 88% client retention in the first quarter of its implementation in 2011 [9–11].

In Ethiopia, it was rolled out in the early 2013 as an MTCT elimination strategy in 2020 [12, 13]. However; only 60.6% of HIV positive pregnant and lactating women were enrolled to HIV chronic care in 2015 [13]. Mothers were still faced a challenge to retain in the service. As evidenced from North East Ethiopia, 16% LTF was documented in which 11.9% was reported at 6 months, 15.7% at 12 months and 22.6% at 24 months follow up period [14]. Similar evidence was reported from Eastern Ethiopia in which LTF was 9.8 and 14.8% at 6 and 12 months respectively [15]. Facility based studies revealed that poor service quality was the main challenge. As an input quality item, some health facilities were made operational with supply chain related problems. Besides, inadequacy of trained human resource was an input related barrier that renders service quality [13, 16]. The lack of service integration, poor adherence to service standards, prolonged waiting time, and confidentiality issues were factors that affect process service quality [16–18].

**Fig. 1:**
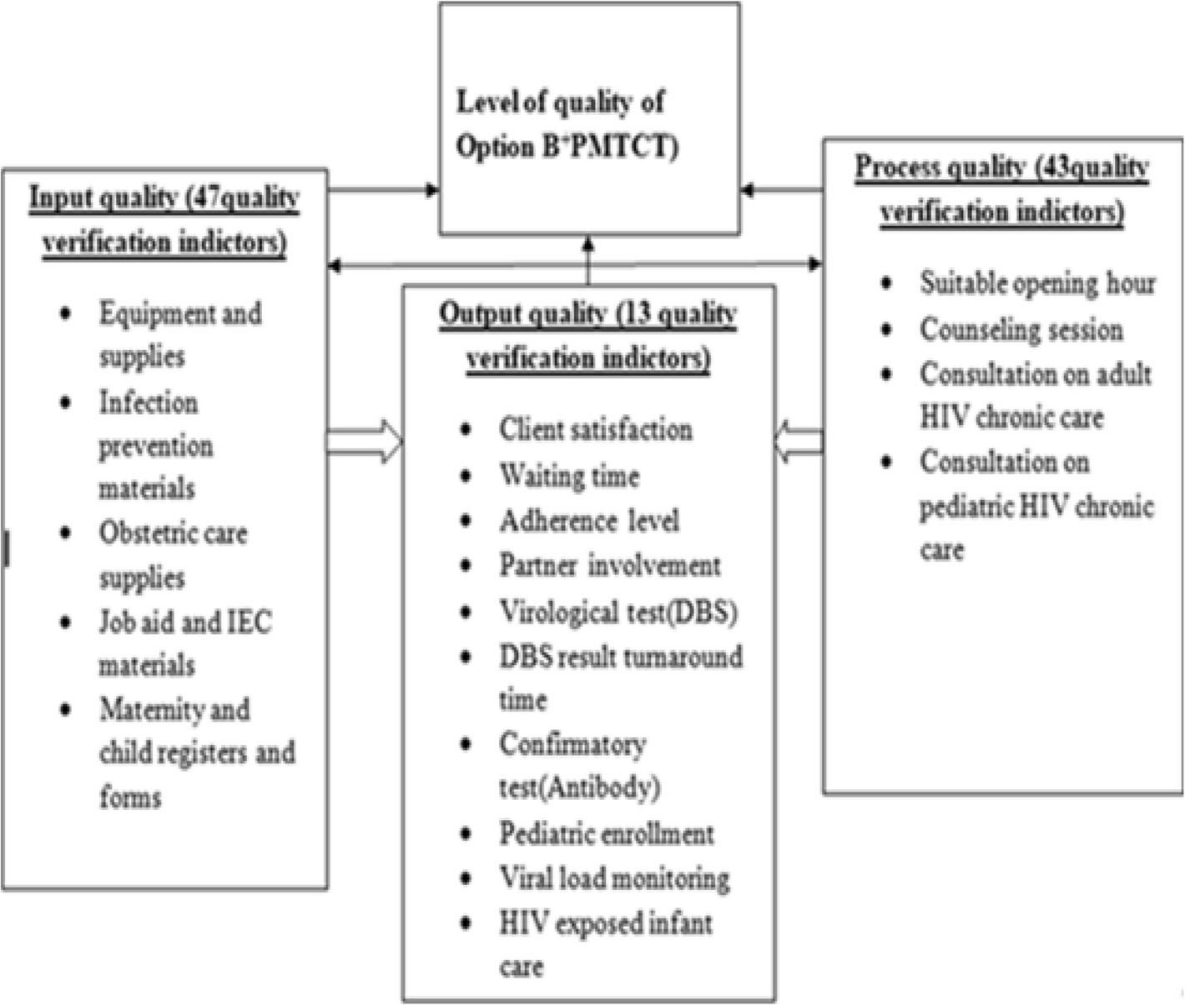
Conceptual framework for assessing quality of Option B + PMTCT in studied health facilities in Mekelle Zone, Northern Ethiopia [19, 20]

Improving service quality of Maternal and Child Health services including Option B+ was a priority agenda in the Health Sector Transformation Plan (HSTP) of Ethiopia to achieve the three 90’s (90–90-90) in 2020 [13]. The barriers reported that affect quality service in the previous studies were input-process-output related factors. As a result, we preferred to use Donabedian model of quality assessment framework to evaluate the service quality [19]. National technical guideline to ensure the provision of Option B+ was also developed based on the three quality components as depicted in the figure (Fig. 1) [20, 21]. Therefore, this study aimed to assess the level of quality of Option B^**+**^PMTCT service provision in public health facilities in Mekelle Zone, Northern Ethiopia using Donabedian Model.

## Methods

### Aim

The study aimed to assess the level of quality of OptionB^+^PMTCT and to explore reasons for good and bad service quality.

### Study design and settings

This study was a facility based cross-sectional survey conducted between February to April 2016. Mixed method approach was used involving both quantitative and qualitative data collection methods. Donabedian model was used [19] as depicted in the figure below (Fig. 1).

The study was conducted in Mekelle zone, Tigray of Northern Ethiopia, 802KMs from Addis Ababa, the capital city of Ethiopia. It is among the top three high HIV prevalence areas in Tigray region [22]. According to the projected national census in 2018 [23], the total population was projected to 320,000. A total of 12 health facilities have been provided Option B+ under MNCH continuum of care. Of which three of them were in hospitals.

### Sampling and sample size determination

All health facilities (nine health centers and three hospitals) providing Option B+ under MNCH platforms were involved in the study. Study participants were HIV positive mother & their infants. They were newly diagnosed women and enrolled under Option B+ package aiming “test and treat” strategy. Besides, who were under continuous follow up for more than one year prior to data collection in MNCH clinic for the purpose of complete clinical history. To come up with the final sample, review of all patient records and follow up charts were conducted which fulfilled predetermined eligibility criteria. First, a total of 219 HIV positive mother & infant pairs were reviewed. However, 48 study participants were excluded since they were transferred from other facilities and did not have a complete clinical history. Similarly, three newly diagnosed HIV positive women spent only six months in HIV chronic care were not included in the study. Hence, the total participants enrolled for the study were 168.

Convenience sampling method was used to recruit participants for qualitative study, until information saturation was obtained [24]. Observation and interviews were conducted during & after service consultation. A total of 60 sessions were involved for direct observation. Of which, 30 HIV positive women and 12 service providers were subjected for exit and in-depth interview respectively to identify reasons for good and bad service quality in respective quality components. Health workers have been providing Option B + PMTCT in MNCH clinic for more than one year were included in the study.

### Data collection and measurements

#### Quantitative data

Quantitative data were collected by four midwives and master’s level supervisor who had an experience in public health data collection.

#### Input service quality

For input quality, 47 indicators were adopted from the national guidelines [20, 21]. Facility inventory was conducted to ensure the availability of essential equipments, drugs and supplies. See list of input quality verification variables (Additional file 1).

#### Process service quality

A total of 43 process related indicators were articulated for assessing process quality [16, 17, 25]. Non-participant observation was conducted using a checklist to observe service adheres to standard practices. The intention was to observe 10 sessions from each facility during each round of data collection. However, this was not possible since too few women came for Option B^+^PMTCT services during the data collection period; indeed, 60 clients in which five for each facility were participated. In addition, some process related variables were obtained from recorded review. See list of process quality verification variables (Additional file 1).

#### Output service quality

Output quality was assessed using 13 items adopted from national guideline [20, 21]. Data were abstracted retrospectively from logbooks and/or records and individual medical records for 168 mother infant pairs using a data abstraction tool. Besides, 12 HIV exposed infant follow up charts were reviewed about the utilization of the Option B+ service package in MNCH clinic. Confidentiality around patient records was protected; communication was made with only authorized person. Patient codes were used and documents were locked. See list of output quality verification variables (Additional file 1).

Overall service quality was assessed by combining input, process, and output service quality items. Facilities were categorized rendering good input service quality, if the average weighted score of input quality performance standards is 100% [10], and 90% or more for process, output, and overall quality performance standards [10, 20]. See the score of each variable for respective quality components (Additional file 1).

#### Qualitative data

Qualitative data collection was guided by principal investigator (corresponding author) who had an experience on qualitative data collection. Both interviews were conducted in Tigrigna (local language), and audio recording was involved.

##### Exit -interviews (EI)

Exit interviews were conducted with mothers who were provided oral consents for participation at the end of service consultation. After 30 interviews were involved with clients, information saturation was obtained; no further interviews were conducted since no new information was being generated. Interviews were guided by using a pre-tested flexible interview guide with probes to identify client views on service utilization. Interviews were lasted within the range of 30–40 min.

#### Key informant interview (KII)

In-depth interviews were held by appointment with service providers during the study. Twelve key informant interviews were conducted until information saturation was obtained. The interviews were guided by using a pre-tested, flexible interview guide with probes to identify their role in providing PMTCT services and reasons for good and bad service quality. Interviews were lasted within 15–25 min.

### Data quality assurance

To enhance data quality, training was provided for data collectors and supervisor with the objective of the study, the nature of the data collection tools, and ways of approaching during interview, observe, record and chart review. During the data collection period there was a strict supervision scheme. Completed questionnaires were checked on a daily basis by supervisor and principal investigator.

### Data management and analysis

**Fig. 2:**
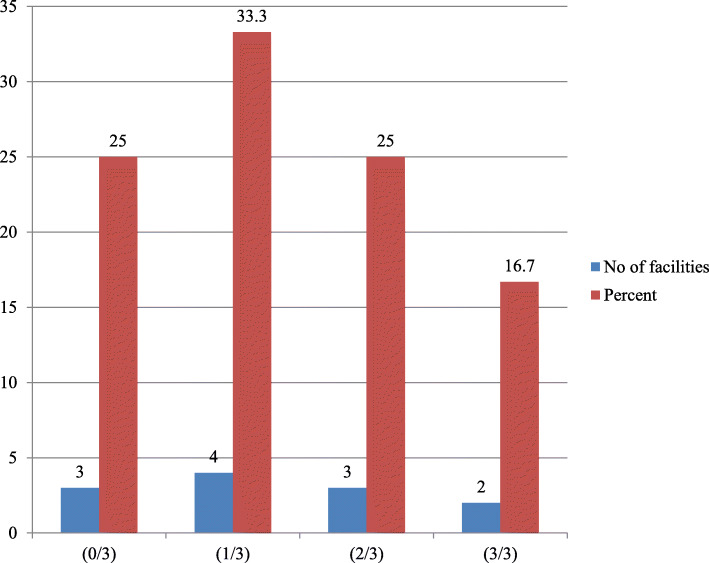
Summary of Option B + PMTCT service quality in studied health facilities in Mekelle Zone, Northern Ethiopia. [Note: (0/3): number of facilities not achieved any of the three quality components; (1/3): number of facilities achieved any one of the three quality components; (2/3): number of facilities achieved two of the three quality components; (3/3): number of facilities achieved all three quality components]

Quantitative data were coded, cleaned, and entered into EPI info version 7 and then exported and analyzed using SPSS version 21 software. Univariate data analysis was conducted to estimate the prevalence of variables for respective quality components. The Findings were presented in tables (Tables 1, 2, 3 and 4) and figure (Fig. 2).

**Table 1:** Health facilities not fulfilling 100% of input service quality performance verification indicators in Mekelle zone, Tigray, Northern Ethiopia (*n* = 12)

Input quality items	No of facilities	Percent
**Human resource and infrastructure**		
Well ventilated waiting room	8	66.7
Well ventilated counseling room	8	66.7
Cleanness of counseling rooms	6	50
Trained service providers on OB^+^	6	50
**Medical supplies**		
Cotrimoxazole prophylaxis	8	66.7
DBS sample collection kit	7	58.3
**Job aid IEC materials**		
PMTCT broachers	7	58.3
PMTCT leaflets	5	41.7
Technical guide line	7	58.3
PMTCT cure card	8	66.7
**Patient forms and registers**		
Referral slips	8	66.7
Referral linkage slips	8	66.7
Appointment cards	8	66.7

**Table 2:** Health facilities not fulfilling 90% of process service quality performance verification indicators in Mekelle zone, Tigray, Northern Ethiopia (*n* = 12)

Process quality items	No of facilities	Percent
Facility suitable opening hour	8	66.7
Client greeting and welcoming	7	58.3
Introducing himself to clients	7	58.3
Waiting time to the counselor	6	50
Adequacy of counseling session	6	50
Counselor confidence during counseling	7	58.3
Conduct history taking	8	66.7
Conduct physical examination	8	66.7
Screening for opportunistic infection	8	66.7
Discus issues of reproductive health	8	66.7
Support for disclosure	7	58.3
Reviewing need of partner notification	7	58.3
Reviewing ARV drug adherence	7	58.3
Reviewing about safe sex practice	8	66.7
Reviewing of HIV infection	8	66.7
Screening for substance abuse	6	50
Discus issues of psychosocial support	7	58.3
Counseling for nutritional support	8	66.7
Screening for STI	8	66.7
Screening for cervical cancer	8	66.7
Calling clients by name	6	50
Encouraging women to ask questions	6	50
Reviewing mothers understanding	6	50
Conduct child growth assessment	7	58.3
Review issues of child immunization	8	66.7
Reviewing issues of infant feeding	8	66.7
Initiating cotrimoxazole therapy	9	75
Review TB risk assessment	8	66.7
Conduct virological test at 6 weeks of age	6	50
Conduct anti-body test at 18 months of age	6	50

**Table 3:** Health facilities not fulfilling 90% of output service quality performance verification indicators in Mekelle zone, Tigray, Northern Ethiopia (*n* = 12)

Output quality items	No of facilities	Percent
Client satisfaction per standard	7	58.3
Clients with good treatment adherence	8	66.7
Clients involved partner testing	5	41.7
Early infant diagnosis for virological test	6	50
Confirmatory antibody test	7	58.3
DBS result turnaround time per standard	4	33.3
Enrolling HIV positive pediatrics to HIV chronic care	8	66.7
Perform CD4 count as base line during their initial visit	7	58.3
Perform CD4 count at least one as follow up visit	6	50

**Table 4:** Summery of themes (TMs) and categories (CAs) developed based on KIIs data in respective quality components in Mekelle zone, Tigray, Northern Ethiopia

Themes (TMs)			Categories (CAs)	
Code	Name	Code	Name	
TM1	Input service quality	CA1	Reasons for good input quality	Good partnership
		CA2	Reasons for bad input quality	Resource constraint
TM2	Process service quality	CA3	Reasons for good process quality	Service integration
				ART initiation regardless of CD4 count
				Simplicity of ARV drug regimen
		CA4	Reasons for bad process quality	Poor service compliance
				prolonged waiting time
				Work load
TM3	Output service quality	CA5	Reasons for good output quality	Patient retention
		CA6	Reasons for bad output quality	high DBS result turnaround time

Qualitative data were analyzed manually using the content thematic approach [24]. Data analysis was done by the first author in collaboration with a second author (experienced qualitative researcher). This involved reading script several times, translating transcripts from local language (Tigrigna) to English, identifying themes (TM1-TM3 …) and categories (CA1-CA6). The main study themes were fitted with three quality components, whilst categories were motivators and barriers for good and bad service quality in each identified themes. All authors were involved in discussions of study themes, & categories. This process facilitated researcher triangulation. Direct quotations were presented reflecting reasons for good and bad service quality.

### Operational definitions

**Input dimension**: this dimension was used to assess the availability of human resources, materials, drugs, equipment, and supplies needed for Option B^+^ PMTCT service provision [17].

**Process dimension**: this dimension used to reflect how service providers adhere to service standards during a service consultation of Option B^+^PMTCT service in MNCH unit [18].

**Output dimension**: used to evaluate the ultimate service result of Option B^+^PMTCT service and patient satisfaction level [17].

**Overall quality**: this particular dimension was determined by combining predetermined three quality components; input, process, and output [25].

**DBS result turnaround time:** The date for DBS sample collection from HIV-exposed infants to the date when HIV screening results was arrived at health facility [26].

## Results

The study was assessed based on Donabedian input-process-output service quality assessment model. A total of 12 health facilities (9 health centers and 3 hospitals) were included which has been providing Option B^+^PMTCT under MNCH continuum of care.

### Quantitative component

#### Overall service quality

The study showed that the overall level of service quality of Option B^+^PMTCT was rendered as good in one out of six(16.7%) of studied health facilities. Specifically, input service quality was judged as good in 33.3% of health facilities, but only 25% of them realized good process and output service quality respectively (Fig. 2).

#### Input service quality

Regarding input service quality, the study revealed that the majority of the health facilities were equipped with clinical care supplies and drugs for Option B^+^PMTCT service provision. Long life ARV regimen (TDF + 3TC + EFV), and other basic obstetric care supplies for Option B^+^ were not reported as stock out for the past one year (additional file one). However; critical input related items for the Option B+ service provision were missed in considerable no of studied health facilities. Only, half of the health facilities kept on hand the necessary trained service providers, drugs for opportunistic infections, and DBS test kits necessary for the desired input service quality (Table 1).

#### Process service quality

With regards to process quality, some prominent key interventions had been missed during service consultation. Option B+ ARV drug adherence counseling and partner notification were offered in 58.3% of the health facilities. The women were greeted on arrival only in 58.3% of the health facilities which had an impact on poor quality service. Prolonged waiting time was also an issue observed during a service consultation [10]. It had been noted that health service providers in the majority of health facilities were observed not adhered to service standards while providing service consultation (Table 2).

#### Output service quality

As an Option B + PMTCT service output, majority; 91.7% of mother infant pair were alive and in their first line recommended treatment regimen in the past one year. However; high DBS result turnaround time and low patient satisfaction level were vital issues which needed great attention while the service has been provided (Table 3).

## Qualitative component

### Input service quality (TM1)

#### Reason for good input service quality (CA1) (Table 4)

The majority of service providers recognized that building team work among program directors and district level experts enabled them to identify availability related factors on time. This is an identified contributing facilitator for facilities to be judged providing good input service quality (Fig. 2). This was illustrated clearly by the following service provider:

**“ … ...***we have been conducted weekly meetings with program managers and district level experts with availability related factors and prepare an action plan to resolve input related constraints on time***”***(PMTCT service provider ≠12 ).*

#### Reasons for bad input service quality (CA2) (Table 4)

Health workers expressed their opinion on shortage of trained human workforce and supply chain issues for Option B+ as a barrier for input related factor. Sometimes, trained staffs were also preferred to serve health care services other than MNCH unit as described below:

***“ … …****Imagine only two health care providers trained on Option B*^*+*^*and serving more than an average of 80 clients per day. Having this reality, how can we provide quality? Therefore, without allocating an appropriate number of trained health care providers , only integrating the service to MNCH unit may not be successful****” (****PMTCT service provider ≠4****)****.* This finding is consistent with quantitative evidence summarized in (Table 1).

**“ … …***Some drug list used for opportunistic infection such as co-trimoxazole prophylactic therapy was reported as stock out for more than six months in the past year lack of transportation was a reason given to us when requested***”***(PMTCT service provider≠8 ).*

### Process service quality (TM2) (Table 4)

#### Reasons for good process service quality (CA3) (Table 4)

Task shifting to scale up Option B^+^ by integrating the delivery of Option B^+^ ART initiation as one service packages in MNCH unit, initiation of ARV regardless of CD4 count, and simplicity of ARV regimen was greatly appreciated by majority of service providers and clients during an interview:

**“ … ...***discrimination is not my concern for the past two years after the adoption of Option B*^*+*^*. I am confident enough to attend my follow up visit together with HIV negative mothers in MNCH clinic. This is because; we all received our follow up care in one room and with the same health professionals****” (****PMTCT client ≠ 18).*

***“****… … Before the introduction of Option B*^*+*^*PMTCT high lost (3%) and dropout rate (4%) was documented in our facility. The main reason forwarded by the majority of the clients was repeated appointments for CD4 count for ART initiation but after its adoption, patient high patient retention was documented****”****(PMTCT service provider ≠ 2).*

**“ … ...***the drug provided for me during PMTCT visit was comfortable and easy to use. I selected a fixed time at 7:00 PM and I am taking the drug usually with a specified time and I don’t want to miss even a fraction of seconds****”****(PMTCT client≠ 21).*

***“ … …****During the time of Option A and B, multiple ARV drugs were prescribed and patients were complaining about the situation, but now patient were easily adhered to the regimen and no more need of continuous adherence support****”****(PMTCT service provider ≠5).*

#### Reasons for bad process service quality (CA4) (Table 4)

The majority of service providers had good experience regarding Option B^+^. However, one health care provider reported her experience of considering CD4 count as criteria for initiating ART which resulted poor service compliance with service standards. Some other providers criticized its integration as creating workload and prolonged waiting time as described as follows:

**“… ...***I am not aware of prescribing ARV drugs regardless of CD4 count and I appointed two PMTCT clients for CD4 investigation before prescribing the drug****”****(PMTCT service provider ≠10 ).*

***“… ...****before the introduction of the Option B*^*+*^*mother living with HIV were under follow up at ART clinic but now they had been enrolled in the MNCH clinic during their maternal and child health care visit which resulted additional work load in our health facility****”****(PMTCT service provide≠3).*

***“… ...****my great concern during my PMTCT follow up visit was an issue of timing to get the service on time since there was delayed service as a result, I have been thinking to miss the opportunity****”(****PMTCT client ≠19).*

### Output service quality (TM3) (Table 4)

#### Reasons for good output service quality (CA5) (Table 4)

As described by majority of service providers, client’s belief in the efficacy of ARVs, absence of stigma and discrimination were facilitators for high patient retention as articulated below:

***“… ...****before the introduction of option B*^*+*^*high patient lost and drop out were documented but now Option B*^*+*^*was highly accepted by patents****”****(PMTCT service provide≠5).*

#### Reasons for bad output service quality (CA6) (Table 4)

Big issue forwarded by almost all participants was high turnaround time for DNAPCR virological test result communication which was arrived within 4–6 months time period of the health facility from the central testing unit that can hinder the success of early infant diagnosis as explained below:

**“… ...***I am always worried regarding delay of my new borne baby’s HIV virological test result. As you have seen, am receiving exposed infant test result today after six months. Unfortunately, I am very much happy today since his result non-reactive. But the past six months were painful for me****”****(PMTCT client ≠28).*

**“… ...***I am always communicating using mobile phone with laboratory experts in the central testing unit an issue of DBS result delay but they told me that the machine was under maintenance” (PMTCT service provider≠ 7).*

## Discussion

The primary aim of this study was to evaluate the level of quality of option B^+^PMTCT and to explore factors for good and bad service quality in Mekelle Zone of Northern Ethiopia. The evaluation was conducted based on three quality components suggested in the Donabedian model and under the umbrella of the national guidelines for Option B^+^PMTCT service provision in Ethiopia [20, 21].

Accordingly, the study result showed that the overall level of service quality of Option B^+^PMTCT was rendered as good in one out of six(16.7%) of studied health facilities. Specifically, 33.3% were judged as providing good in terms of input quality but only 25% for the process and output service quality respectively. However, it’s important to note that the three quality components are interlinked to each other and the effect of one component had its own impact on the other [19].

This finding was by far from pre-determined national target in 2020 [13] and evidences from Southern Ethiopia [16, 27]. The reason for such discrepancies might be due to the stretched nature of the second health sector transformation plan and methodological difference in which only client interview was conducted to assess client satisfaction in the latter two studies.

Regarding input quality, it was found better provided than its counterparts. The study showed that only, 33.3% of the studied health facilities had the necessary inputs to provide quality delivery service. It was relatively lower when compared to some other African countries [9, 28, 29]. This discrepancy might be due to variation in national guideline and performance targets that lead to variation in service quality. However, the study revealed the finding was almost consistent with a report from Northern and South West Ethiopia [18, 25]. From the qualitative finding, absence of continuous and timely inventory of resources at facility level lead shortage of input related resources as described by key informants from Southern parts of Ethiopia [27]. This had an impact primarily on input quality [19].

The study also revealed that only one fourth of the study health facilities fulfilled service adherence to process related service delivery of Option B+ according to the national guideline. This finding was comparable with evidence from Northern and South West Ethiopia [18, 25]. But lower when compared with evidence from Malawi [30]. A variation of the finding in the latter study might be due to their good experience in implementing the service since it was an area in which Option B^+^PMTCT first piloted internationally [9]. Identified barriers for process related factors during an interview were poor service adherence to service standards, work load, and prolonged waiting time which were similarly reported from [27, 31, 32]. This process related factors had serious implications in compromising the overall quality of care currently aspired in 2020 [13, 19].

Similar to process quality, the study showed good service quality was archived in one fourth of the studied health facilities in terms of service utilization and satisfaction of mothers for Option B^+^PMTCT. Patient satisfaction is one of the desired items in the measure of output service quality [19]. The overall client satisfaction about the service was reported low (58.3%) which had an impact on service output quality. Qualitative finding suggested that high turnaround time for DBS result communication and prolonged waiting time to get the service genuinely forecasted that significantly affect service output. This finding seems very plausible and significantly associated with other evidences [33–36].

## Conclusions

The study revealed that health facilities that achieved the aspired level of service quality were insignificant in number. Only, 16.7% of study health facilities fulfilled the desired level of quality based on predetermined quality judgment criteria in each quality components. To realize the current aspired level of service quality in the country’s health sector transformation plan, comprehensive program monitoring is needed based on three quality components in line with identified reasons for good and bad service quality to improve the overall service quality. This was due to their interrelated effect of one another [19]. Good partnership, service integration, ART initiation regardless of CD4 count, simplicity of ART regimen were facilitators positively influencing the quality of care; whilst, resource constraints, poor service compliance, prolonged waiting time, workload,and high DBS result turnaround time were barriers negatively affecting the service quality.

### Study limitations

The model used in this study had its own drawbacks that considered only linear assumption that do not infer casual relationships. In measuring process quality, overestimations of findings could happen due to either Hawthorn or social desirability biases during service observations. Use of small sample size may affect finding generalizablity but in process evaluation generazablity is not an issue. In addition, the rigor statistical test was not done since it was program based process evaluation and use of sophisticated statistical analysis is not a must unlike outcome evaluation [37].

## Supplementary information

**Additional file 1.** Excel files showing the detail scores for the three quality components.

## Data Availability

The datasets generated and analyzed during the current study were not publicly available due to the nature of sensitive data on HIV/AIDS and confidentiality issues. The data can be available upon reasonable request to the corresponding author.

## Abbreviations

MTCT: Mother to Child Transmission
DBS: Dried blood spot test
PCR: Polymeric Chain Reaction
HIV: Human -Immune -Virus
MNCH: Maternal, Neonatal, and Child Health
ART: Antiretroviral Therapy
ARV: Antiretroviral Medication
PMTCT: Prevention of Mother-to-Child Transmission
WHO: World Health Organization
KII: Key Informant Interview
TM: Themes
CA: Categories
